# Clinical Researches on Auscultation of the Respiratary Organs, and on the First State of Phthisis Pulmonalis

**Published:** 1842-04-01

**Authors:** 


### Clinical Researches on Auscultation of the Respiratory Organs, and on the First Stage of Phthisis Pulmonalis. By *Jules Fournet*, Paris. Translated from the French by *T. Brady*, M.D. Fellow, and Professor of Medical Jurisprudence, in the Dublin College of Physicians. Part I. London. Churchill.

M. Fournet's work consists, in the original French, of two parts; the first containing his Researches on Auscultation of the Respiratory Organs, and the second, his Researches on Phthisis Pulmonalis. Though the principal aim of the author has been to establish the diagnosis of phthisis in its earlier stages; this first part will be found to form a tolerably complete system of auscultation of the respiratory organs, * "in which, while some errors, sanctioned by the authority of Laennec, are rectified, and some omissions supplied, most of the physical phenomena are considered in a point of view somewhat different from that in which they were contemplated by the great discoverer." It is not a little extraordinary, nor, indeed, a little complimentary to the genius, sagacity, and persevering industry of Laennec, that the science of stethoscopy, instead of growing up slowly and gradually, and becoming matured under the fostering care of his successors, should have come forth from his brain, as Minerva came from the brain of Jove, a full-grown and adult maid, armed cap-à-piè, and perfectly prepared for the discharge of the duties of her assigned mission.

* Translator's Preface.

True it is, that, from time to time, various little additions have been made, and some rather hasty generalizations of Laennec have been corrected, or at least restricted; but, if we remember, that the individuals who ventured on introducing these improvements, religiously followed in the footsteps of the great original himself in so doing, never swerving either to the right or to the left, we shall be disposed to consider such improvements as donations or tributary offerings to this offspring of their master in acknowledgement of her all-perfection and undisputed sway, rather than as by any means implying any thing like a deficiency or imperfection in her congenital endowments.

M. Fournet, however, in a spirit, certainly not of gallantry, rather of knight-errantry, which some of Laennec's old and attached followers will be disposed to designate Quixotic, has boldly come forward to dispute the pretensions of the lady, and to question her right to her so-long recognised authority. Whether M. Fournet was actuated to this bold and adventurous enterprise by his conviction of the truth of the pathological law, that precocious children are in general rickety, we know not. We shall now, however, proceed to see what he has to say for himself. He has divided his work into two parts, the first containing his researches on auscultation; the second, those on phthisis. With respect to the subject of the first part, he says, that the analysis of the two murmurs of respiration, the inspiratory, and the expiratory murmur, instead of the single murmur, as described by Laennec, has multiplied the number of signs by means of which we recognise the diseases of the respiratory organs, and has dissipated some errors which followed necessarily from the consideration of a single murmur, instead of the two that really exist.

The analysis of a greater number of the properties of sound, than Laennec noticed, has enabled him to ascertain a greater number of morbid modifications of the respiratory murmurs, to establish new relations between the symptomatology and pathological anatomy, and to multiply, by this new means, the signs of pulmonary disease. The determination of the law of co-existence of the morbid sonorous phenomena of the respiratory apparatus with inspiration or expiration, has served also to increase the number of these relations and signs.

Our author tells us that, on listening attentively to the respiratory murmur in persons with healthy and well-formed chests, he was astonished to hear two sounds, instead of the single one described by Laennec. On prosecuting the enquiry, he felt satisfied that, in the normal state, *inspiration and expiration were each accompanied by a very distinct murmur;* the latter being merely much weaker than the former. This division of the single sound, named by Laennec the respiratory murmur, is further confirmed by pathological observation, as the weaker sound is found to become at times the stronger, and *vice versâ*.

The fact of the existence of the two murmurs being established, their morbid modifications being also clearly ascertained, as well as the existence of a number of signs corresponding to these modifications, it is necessary, in order not to lose any of those signs, to investigate in the normal murmurs all the different circumstances from which they may be derived. By this means the number of signs will be increased, and the science of auscultation will gain both in extent and in precision; in extent, by the appreciation of a greater number of characters, both normal and morbid, and by the application of these characters, to two sounds instead of one; in precision, by the more rigorous analysis of these characters, by the greater facility of comprehending the chain of their relations, and the general principles which govern them.

The following are the fundamental characters which the author deems it useful to analyse in the respiratory murmurs :—

1. The proper or distinctive character. 2. The hard or soft character. 3. The dry or humid character. 4. The quality (*le timbre).* 5. The tone. 6. The intensity. 7. The duration. 8. The rhythm. All the morbid modifications of the respiratory murmurs, capable of any practical application, the author refers to one of the above sources.

After attempting to explain the meaning he attaches to these different characters, he shows that they may be combined with each other in a thousand ways, and that the diagnosis may be most materially aided by the analysis of these combinations. For the purpose of illustrating the nature, mode of production, morbid modifications, and semeiological import of these several characters, more especially of the dry, humid and bubbling characters, he adduces some very ingenious experiments with sponge, the different physiological and pathological conditions of the lung being capable of being tolerably well represented by the sponge suitably adjusted. The same experiments serve also to illustrate the mode of production of the inspiratory and expiratory murmurs and their relation to each other.

"The murmur of inspiration and that of expiration," says M. Fournet, "marking each a separate period in the complete movement of respiration, the different morbid sounds may present themselves with the one or the other only, or with the two together; and as the causes on which this co-existence depends are usually physical causes, fixed causes, it follows that the co-existence of the morbid sounds with such or such a period of respiration, may, in many cases, furnish useful diagnostic signs, because we are in this way enabled to ascend from the effect to the cause."

Dr. Jackson, a young American physician, and who had prosecuted his studies in the Parisian hospitals with considerable eclat, was among the first, if not the very first, who made mention of the expiratory murmur. What he has said of it, however, refers to its morbid form, it having been first observed by him in a case of tuberculated lung. This occurred as far back as 1832. Several other pathologists also, among the rest Andral and Louis, have recognised the presence of the expiratory murmur both in the healthy state, as well as indicative of certain morbid conditions of the respiratory organs. Dr. Cowan also, in the notes appended by him to his translation of Louis on Phthisis, more than once points out the importance of the expiratory murmur, as a means of diagnosis in the very early stage of that disease. It is rather strange that the existence of the expiratory murmur was recognised in pathology, before it was even suspected in physiology; this circumstance, however, can be readily explained from the fact of its being much more developed, and, consequently, much more appreciable in the former than in the latter. Our author, after giving certain precepts for the practice of auscultation, in which, however, there is nothing novel or extraordinary, proceeds to—

### CHAP. I.—ON THE PHYSIOLOGICAL SONOROUS PHENOMENA OF RESPIRATION.

He first considers each section of the respiratory apparatus separately, and investigates—1st, the characters of the inspiratory and expiratory sounds in each; 2nd, the successive alterations these sounds suffer, the differences they present in these different sections; 3rd, the relation between these alterations, and the differences in texture of the parts where they originate; and 4th, he proposes to enquire whether the sounds produced are exactly the same on both sides.

*Inspiration.*—In the vesicular section, the proper character of the inspiratory sound is a light *breathing*, or *blowing;* perfectly pure, without any admixture of an accessory sound; the author objects to the epithet *vesicular* being applied to this murmur in its natural healthy state. The duration and intensity of the inspiratory murmur are two characters which should be well appreciated in consequence of their symptomatological value. The expiratory, like the inspiratory murmur, is represented in its proper character by a pure and gentle murmur. For the purpose of avoiding the inconvenience of a *nominal* valuation, which cannot serve as a unit of measure sufficiently exact to enable us to compare with it the modifications these characters undergo, he has adopted a numerical valuation of them, selecting the numbers 10 and 2 to express the comparative intensity and duration of the inspiratory and expiratory murmurs; viz. the intensity and duration of the inspiratory bearing the same ratio to those same characters of the expiratory murmurs, as 10 bears to 2. Though, the numbers 5 and 1 would express the same ratio, he prefers the larger numbers, as affording him a longer scale of increase and decrease above and below these two extreme limits. He next considers the bronchial, tracheal, laryngeal, pharyngeal, &c. sections; and first,

### *Normal Bronchial Respiration, and the differential diagnosis between it and morbid Bronchial Respiration.*

The only part, generally speaking, where the normal bronchial respiration can be heard is the part of the posterior region of the chest corresponding to the root of the lungs. The normal bronchial respiratory murmur differs from the morbid bronchial, 1st, in its intensity, which is much less; 2nd, in its duration (shorter); 3rd, in its *quality*, which is much less marked; 4th, in its exclusive and very circumscribed seat at the root of the lungs; 5th, in its co-existing more peculiarly with the period of expiration, while the morbid bronchial character, at a somewhat higher degree, co-exists equally with both periods.

Our author next cautions the stethoscopist against mistaking the buccal, pharyngeal, or nasal sounds for the bronchial respiration, and then considers the question, whether the respiratory sounds are the same in the two sides of the chest, and in the different regions of the same side? From numerous examinations made by him he is satisfied that, in the normal state, the respiratory sounds are equally audible on both sides of the chest, and *that whenever we find a difference between the murmurs at the summits*

*of the lungs, this difference may in general be attributed to a pathological condition.* This question, thus peremptorily solved by M. Fournet, is one of great practical importance; Dr. Stokes entertains on this point an opinion directly the reverse; his experience leads him to think that in many cases there is a natural difference between the intensity of the murmur in either lung, and that in such cases the murmur of the *left* is distinctly louder than that of the right.

With respect to the normal resonance of the sounds of the heart in the sub-clavicular regions, our author makes a remark of some practical importance; namely, that the mere equality of the resonance of the heart's sounds under both clavicles may, in the majority of cases, be considered an indication of an increase of density at the top of the right lung. This sign may be turned to account in the diagnosis of the first stage of phthisis.

### Chap. II.—The Morbid Sonorous Phenomena of Respiration

Are divided into two classes: 1st, such as are merely modifications of the normal sonorous phenomena; 2nd, such as, not having previously existed under any form, are newly-developed in consequence of certain organic alterations, as metallic tinkling, the pleuritic friction sounds, &c. Again, the modifications in the normal sonorous phenomena of respiration he classes under four following heads: modification by increase—by diminution—by cessation—and by perversion; the modifications by perversion establishing a sort of transition or connecting link between the two classes. The various ronchi, which Laennec considered as foreign to the sounds of respiration, M. Fournet considers as being only modifications of certain normal characters of the respiratory sounds. The modifications by augmentation or diminution do not affect the nature of the respiratory murmurs; they affect only their intensity and duration. The changes in the nature of the murmurs belong only to the class of modifications by perversion. This perversion may affect either the *quality*, the soft mellow character, the dry, humid character, or the rhythm. The alterations the respiratory sounds suffer in their *quality* are numerous. There are, in these alterations of *quality*, successive degrees, which lead by a considerable number of gradations, from the most simple to the most elevated forms. They are, as it were, so many degradations of a common type, which recals the sensation of metallic *quality*, and which may be designated by that name. The signs which Laennec has described separately under the names of *cavernous, blowing, amphoric* respiration, and under that of *metallic tinkling*, all belong to this type. They all give to the ear a sensation of something metallic; only this sensation is produced by each in a different degree: we see them in certain diseases follow one another in order, and sometimes appear in succession from the first shade to the last. From the moment the first shade of bronchial character appears, till amphoric resonance or metallic tinkling strikes the ear, there is an unbroken, but graduated chain; a successive passage from one degree to another, but no change of nature. The following is the scale of these degradations: 1, metallic tinkling; 2, amphoric character; 3, cavernous character; 4, bronchial character; 5, blowing character; 6, resonant character; 7, clear character. Two physical conditions, either single or united, are found to exist in all cases, where the metallic quality is manifested; viz. condensation of the pulmonary tissue, and increase in the diameter of the air-tubes.

*Expiratory Murmur.*—Augmentation of this murmur occurs under two different forms: in the 1st the normal ratio between the inspiratory and expiratory murmurs is maintained; in the 2nd this ratio is destroyed. In the first case the inspiratory sound has increased in the same proportion as the expiratory; in the second, the inspiratory murmur has remained in its normal state, or has even diminished, while a progressive elevation has taken place in the expiratory. Supplementary respiration is an example of the first; the second is seen in the first stage of phthisis, and in emphysema of the lungs, these being the only affections in which increase of expiration with decrease of inspiration are met with in a high degree; hence the value of this double diagnostic in these two diseases.

After instituting a parallel between the morbid character of the inspiratory and expiratory murmurs, our author next goes on to consider the relations, physiological and morbid, between the respiratory murmurs and movements. This point he endeavoured to ascertain by physiological and clinical experiments. From these, he felt himself warranted in inferring that there exists between these three facts—1st, the intensity and duration of the respiratory murmur; 2, the frequency and extent of the thoracic movements; 3, the degree of the expansive and locomotive force of the lung—so intimate a connexion, that the derangements of one necessarily re-act on the other, and hence, that we may, from those derangements that can be seen or heard, appreciate those that cannot directly be observed during the patient's life. The connexion, however, between these three facts is far from being as general or absolute in pathology as in physiology.

After considering the morbid characters of the voice and cough, in which part of his work he introduces some modifications of the opinions of Laennec, he next enters into the subject of *Exaggerated Respiration*, sometimes called supplementary respiration, and by Laennec the puerile respiration. The character of exaggerated respiration is a pure and simple increase of the inspiratory and expiratory murmurs, an increase which affects both the intensity and duration of these sounds. The increase in expiration is proportionately greater than in inspiration. The inspiratory murmur, whose normal number is 10, may rise to 12 and 15; the expiratory may attain the same elevation. Under certain circumstances exaggerated respiration may be confounded with certain morbid forms of respiration, whose diagnostic import is quite different. These circumstances are the following:—

1. The increase of expiration that takes place in the first stage of phthisis may be mistaken for mere exaggerated respiration; the following characters will distinguish them:

In supplementary respiration, the increase in inspiration affects both the duration and intensity of the sound—in phthisis, the intensity alone is augmented, and in general the duration is diminished. In the first, the number that marks expiration never exceeds that of inspiration—this often happens in the second. In the inspiration and expiration of phthisis, the character of augmentation is combined with a certain roughness, hardness, and dryness, which are quite foreign to exaggerated respiration. In the first stage of phthisis the bronchial character is sometimes preceded by a slight shade of metallic *quality;* but this does not shew itself in inspiration till after it has co-existed with expiration, whereas the slight clear *quality* of supplementary respiration appears at once in both murmurs. When the alterations of *quality,* which belong to the first stage of phthisis, exist in both inspiration and expiration, they have become too well marked to be confounded with the slight shade of clear *quality* of exaggerated respiration. Besides, in phthisis, the alterations of *quality* continually tend to increase in intensity; in supplementary respiration the clear quality we have mentioned remains always the same. An obscure or dull sound on percussion usually accompanies the respiration of a tuberculized lung; in supplementary respiration the sound is rather increased than diminished. In the first, the usual seat of the morbid characters is the top of the lung; they are localized below the clavicle—this localization is very rare in the other.

2. In emphysema of the lungs there is also increase of expiration, as in exaggerated respiration; but these two morbid states differ essentially in other respects:—in emphysema, the inspiration is diminished at the same time that the expiration is increased. With these characters is joined that of harshness and dryness. The sound on percussion is almost tympanitic.

3. Between exaggerated respiration, and rather strong natural respiration in the adult, there is this difference; that, in the latter, expiration has at the most increased only 1, 2, or 3 degrees, that respiration is the same in all parts of the chest, and that there is not the co-existence of any morbid phenomenon—whilst in the former these circumstances are reversed. The circumstances which may produce exaggerated respiration are, 1st, nervous influence; 2d, external physical influence; and 3d, internal physical influences, which, generally speaking, are organic alterations. In this form of respiration, inspiration returns to its normal state sooner than expiration. This is conformable to the author's general principle, that in all kinds of morbid respiration, when the expiratory murmur is increased, it returns to its normal state more slowly than the inspiratory.

We now come to the description of a sound quite distinct from all those hitherto described—this sound he calls the *pulmonary crumpling sound.* Its general character is, that it gives the ear the sensation of squeezing or crumpling. "It seems," he says, "as if the eye, receiving the same impression as the ear, saw the pulmonary tissue struggling with effort and with noise against the obstacle which obstructs its expansion. This sound, though sometimes very perceptible, seldom extends beyond the point where it is generated. It generally co-exists with inspiration alone; sometimes however it is heard during inspiration and expiration, but more marked in the former. With respect to the diseases in which this pulmonary crumpling sound appears, he has as yet discovered only in the three following circumstances:—1st, in a woman, whose lungs were compressed by a large encephaloid tumour seated in the anterior mediastinum. 2. In a patient, in whom there was found a very large non-tuberculous cavity, containing only air, and bounded superficially by walls two or three lines thick, firm, flexible, and resembling perfectly a piece of leather of the same thickness; in this case the crumpling sound, which was very intense, was produced by the to and fro motion of this wall, caused by the passing of the air in and out of the cavity; in this case too, the sound was heard both in inspiration and expiration. And 3dly. This sound is heard chiefly in the first stage of phthisis. With respect to the site of this sound, it is wherever the anatomical conditions necessary for its production exist; in phthisis it exists at the tops of the lungs only; most usually in front below the clavicle, sometimes behind in the supra-spinal fossa. The anatomical conditions necessary for the production of the crumpling sound, are the following: 1st, a mechanical obstacle to the expansion of the lung; 2, lobular induration of the pulmonary tissue; 3, alternate flapping to and fro of a thick and dense lamina of fibrous tissue. The author found this sound to exist in about one-eighth of the cases of phthisis, in which he noted the physical signs; he accounts for the smallness of this number from the circumstance, that as it exists only during a limited period, it is unobserved in several cases.

With respect to the differential diagnosis of this sound, the phenomenon with which it may be most easily confounded is the dry crackling ronchus, as there are several points of analogy between them; the crackling ronchus however is infinitely more frequent than the crumpling sound, and the impression it makes on the ear is altogether different. The pleuritic friction sound might more easily be mistaken for the pulmonary crumpling. He gives the following as the distinguishing characters: the latter is continuous; in listening to it, the ear feels no sensation of displacement, it is produced, almost exclusively, in the top of the lung; it co-exists with the signs of the earliest stage of phthisis; the patient has usually no sensation from it; the hand cannot appreciate it, and it exists almost exclusively in inspiration. The pleuritic friction sound, on the contrary, is composed of little jerks or shocks; the ear receives the sensation of displacement from above downwards, and from below upwards, according as it is heard during inspiration or expiration; it almost always co-exists with both periods of respiration; it is usually produced in a part (the middle of the posterior part of the chest) where the other is never met; and it is in general appreciable by the hand as well as sensible to the patient.

The next sound to which our attention is directed is the *crackling ronchus, or sound of pulmonary crackling* (râle de craquement, ou bruit de craquement pulmonaire.) The sound differs from all the other *ronchi.* On its first appearance it bears and deserves the name of the *dry crackling ronchus;* after it changes perceptibly to a *humid crackling,* so as to become a mere mucous ronchus; and eventually it becomes the gurgling ronchus. This ronchus is formed by a succession of small sounds; each of these is a crack, and it is the sum of these sounds during one of the respiratory movements, that constitutes the crackling ronchus, The humid ronchus is also composed of several successive sounds, which gradually assume more the form of bubbles, and in the end they present perfectly the bubbling character. The crackling ronchus is found co-existing with inspiration more exclusively in proportion as it is more dry. As it becomes humid, it is heard in expiration also.

The seat of this crackling ronchus is in general the top of the lung, and if it be heard in points remote from this, it is when the signs of softening, or of a cavity have already appeared in the top of the lung. From what has been said it is sufficiently obvious that this ronchus is characteristic of phthisis: the dry crackling ronchus belongs to that period of the first stage of phthisis, which may be called the second phasis; whilst the humid crackling ronchus belongs to the third phasis, and establishes in some degree the transition from the first stage of the disease to the second. From this it appears that the dry crackling ronchus is not the first symptom that appears in phthisis: several of the modifications in duration of the respiratory murmurs precede it.

The anatomical conditions on which the crackling ronchi depend may be thus summed up: the dry crackling ronchus corresponds to that phasis of the first period of phthisis, which is represented by a simple infiltration of crude tubercles into the pulmonary tissue; the humid crackling ronchus appears to be, in its different phases, the exact symptomatic representative of the process of softening which gradually pervades the crude tubercles.

M. Fournet next describes the *humid ronchus with continuous bubbles*, which he has observed to be a prominent sign of *active sanguineous congestion of the lungs*, in which pathological state the diagnostic value of this sign he has found to be very great. He then proceeds to the description of the pleuritic friction sounds, which want of space on our part, as well as want of entire novelty on their part, prevent us from noticing more in detail.

In Chap. III.—The course and connexion of the sonorous phenomena, their relative value, and their different combinations are pointed out very clearly; in this part of the work also œgophony, bronchophony, and pectoriloquy, instead of being three different phenomena, are shewn to be but three successive degrees of the same phenomena. The same is proved of the different morbid sounds that compose the class of alterations of *quality*.

In Chap. IV. we are presented with a classification of the sonorous phenomena, physiological and morbid, of the respiratory apparatus, and with separate tables of the healthy and morbid sounds. Also with a plan of a classification of the several kinds of ronchi founded on their respective characters.

In Chap. V. the author considers the laws of co-existence of the morbid sonorous phenomena of the respiratory system with inspiration or expiration. In these laws will be found a number of differential characters extremely useful in practice. Certain sounds of very different diagnostic value, are sometimes confounded with one another in many respects, and are distinguished by this character, viz. that some co-exist with both periods of respiration, whilst others co-exist with one only; such are, for instance, the humid bronchial ronchi, compared with the vesicular ronchi; whilst there are other signs, which, appearing first in expiration, and not extending till later to inspiration, enable us in this way to estimate the progress of the disease; such are the alterations of *quality*. The constancy, for the most part, of the laws of co-existence of the sonorous morbid phenomena with this or that period of respiration, gives, in general, considerable value to the signs derived from this source. This idea however of employing this method of diagnosis is by no means a new one. Several pathologists have already made allusion to it.

Chap. VI. contains the characters presented by the inspiratory and expiratory murmurs in each of the diseases of the respiratory system considered separately. As this chapter contains several points of a practical bearing, we shall give a succinct summary of it. In this part of his work the author introduces numerous changes in the symptomatology of pulmonary diseases, as given by Laennec; these changes he considers to be perfectly borne out and fully sanctioned by the peculiar method adopted by him in the study of auscultation.

### ART. I. DISEASES OF THE BRONCHI.

*Acute Bronchitis of the large Bronchial Tubes.*—First Stage.—1. Increase of the intensity and duration of the inspiratory and expiratory murmurs, easily recognized, wherever the sonorous and sibilant ronchi are not heard, the increase being greater in the expiratory than in the inspiratory murmur.

2. A little dryness and hardness of both murmurs.

3. Dry bronchial ronchi, both acute and grave, but especially the latter, and heard chiefly in expiration.

Second Stage.—1. Exaggeration of the respiratory murmurs not carried quite so far.

2. The humid and viscid character taking the place of the dry one.

3. Humid bronchial ronchi, bubbles growing larger and larger.

*Chronic Bronchitis.*—1*st, with increased mucous secretion.*—1. Slight decrease of the duration, and still more of the intensity of the inspiratory murmur; increase of both the intensity and duration of the expiratory.

2. Difficulty of production in the inspiratory and expiratory murmurs, especially the former.

3. Humid character of these murmurs.

4. Humid or mucous bronchial ronchi with large bubbles.

2*nd. Chronic Bronchitis, with decrease or suspension of the mucous secretion.*—1. Sometimes a great decrease of the inspiratory and expiratory murmurs, which may fall as low as 1.

2. Character of difficulty of production in both murmurs; character of dryness and hardness.

3. Dry bronchial ronchi, equally audible during both periods of respiration.

*Dilatation of the Bronchi.*—1. Decrease in the intensity and duration of the inspiratory murmur, which may fall to 6 or even to 5. Increase of intensity and duration of the expiratory, which may rise to 12 and 15.

2. All the alterations of quality, from the simple degree of clearness up to the cavernous character, appear in succession, and extend from expiration to inspiration, in proportion as the disease passes from its earliest to its most perfect forms.

3. All the humid bubbling ronchi comprised between the simple mucous ronchus and the cavernous ronchus.

4. Voice bronchophonous or pectoriloquous.

5. Bronchial or cavernous cough.

### Art. II.—Diseases of the Pulmonary Tissue.

*Pulmonary Emphysema.*—The author denies Laennec's accuracy in setting down the friction sound of ascent and descent, and the dry crepitant ronchus with large bubbles as pathognomonic of inter-lobular emphysema, there being, according to him, no symptomatic difference whatever between this and the vesicular form of the affection.

1. Gradual diminution of the inspiratory murmur from 10 to 2, or even 1, affecting both its intensity and duration, with considerable increase of the intensity, and still more of the duration of the expiratory murmur, from 2 to 10, 15, 18, 20.

2. Character of roughness, hardness, difficulty and dryness in both respiratory murmurs.

3. Dry bronchial ronchi, both grave and acute.

4. The transmission of the voice, cough, and of the sounds of the heart through the emphysematous tissue of the lung, more imperfect than in the normal state, and inversely as the degree of the emphysema.

5. Increase of the normal resonance on percussion, with a somewhat tympanitic character in the sound.

6. Diminution of the normal thoracic vibration, as perceived by means of the hand applied to the chest, whilst a person speaks.

7. Rounded form of the chest.

8. Inspiration, abrupt and short, produced by a kind of convulsive movement, in which the entire thorax, as a single piece, is forcibly elevated, the infero-lateral parts of the chest almost alone affecting the movement of dilatation; more or less depression of the supra-sternal fossa and supra-clavicular regions—the mean duration of the movement may be represented by 3.

Expiration seems to be nothing more than relaxation of the muscles previously violently contracted; it is produced by the gradual sinking in of the thorax—its mean duration is 9.

Almost complete abolition of the partial movements of the chest, (movements by which the ribs are separated and approximated,) which no longer moves, either in inspiration or expiration, except by a motion of the whole.

We shall now pass on to the signs of *phthisis pulmonalis* in its first stage, as furnished by auscultation, percussion, &c.

1. Diminution of the duration and intensity, but especially of the duration of the inspiratory murmur, which may fall to 4 and even 2.

2. Augmentation of the intensity and duration of the expiratory murmur, which may rise even to 20.

3. Rough, hard, dry, difficult character in the murmurs, but especially in the inspiratory.

4. Alterations of *quality,* appearing first in expiration, spreading afterwards to inspiration, preserving greater intensity in the former, and passing successively through all the alterations of *quality,* from the clear to the amphoric character.

5. Pulmonary crumpling murmur or ronchus, co-existing almost exclusively with inspiration.

6. Dry crackling ronchus, co-existing almost exclusively with inspiration, and passing successively into the humid crackling, the cavernulous and cavernous ronchi, which occupy more and more equally both inspiration and expiration.

7. Bronchophony more or less intense.

8. Bronchial cough more or less marked.

9. Transmissions of the sounds of the heart through the tuberculated parts of the lung.

10. Sound on percussion over this part more obscure.

11. Diminished vocal vibration.

12. Diminution of the partial movements of the upper parts of the chest; diminution of their volume; sinking in of the spaces above and below the clavicle.

We shall here close our notice of this work, not, however, without expressing our entire satisfaction with the talent and industry displayed by M. Fournet in his clinical researches into a class of diseases, to a complete knowledge of which much is still wanting, notwithstanding the very successful labours of so many distinguished pathologists in this field of science. By directing attention to the two murmurs of respiration he has we feel done good service to the cause of thoracic pathology; one of the first fruits of which will we doubt not be, the power of diagnosticating phthisis pulmonalis, at a much earlier period of this destructive disease than was hitherto practicable.

To Dr. Brady the profession is not a little indebted for his very elegant translation of the work. The style is chaste and classic; and the manner in which the doctor has performed his task, proves him to be perfect master of his subject. We trust that he will ere long favour his medical brethren with the second part.

